# Defending Against Medical Error: Personal Reflections on the Legacy of John Senders

**DOI:** 10.1177/00187208211033414

**Published:** 2021-08-02

**Authors:** Joseph A. Cafazzo, Abigail J. Sellen, Don Norman

**Affiliations:** 1 7989 University Health Network, Toronto, Canada; 2 10438 Microsoft Research, Cambridge, UK; 3 8784 University of California, San Diego, USA

**Keywords:** human error, healthcare, human-centered design, system thinking

## Abstract

**Objective:**

To honor the legacy of John Senders, a distinguished member of the *Human Factors and Ergonomics Society*, by a short, personal history of him, but then to honor his legacy by extending it through our own professional opinions, with an emphasis on the study of human error and its implications for healthcare systems—two topics in which he excelled.

**Background:**

The authors are familiar with the topic and subject matter. One was a friend of Senders for over 50 years. Another was a collaborator and joint author with Senders (as well as his stepdaughter). All three authors have extensive publications in the topic areas.

**Method, Results, and Conclusion:**

The authors used personal accounts of interactions with Senders at conferences, experiences living and working with him, and a brief review of his most personal, notable publications in healthcare. The reflections indicate a strong resonance on Senders’ contributions to system design that are relevant today in healthcare’s most challenging period in its history.

Who was John Senders? The three of us have known him for varying lengths of time. Cafazzo knew about him but had only briefly met him when the three of us attended his 90th birthday celebration. Sellen, John’s stepdaughter, was taught by him and coauthored various papers with him. As for Norman, he interacted with John for over 50 years, starting in Cambridge, Massachusetts in the early 1960s, but bumping into him at odd times and places, most notably at the two “Clambake Conferences on Human Error.” The Clambake Conference was the first such conference ever to be dedicated to the study of human error. John and his wife, Ann Crichton-Harris, held it at their home in Maine in 1980 (where they also struggled to turn an old turbine into a money-making generator of electricity powered by the waterfall across from their property). The conference “dinner” was actually a clambake, held on a sand spit near their house. Three years later, there followed the second “Clambake Conference,” this time at the Rockefeller Foundation’s Bellagio Center in Italy. There was a before-dinner sherry hour, dinner, after dinner drinks, and a music recital by members of the local music seminary with attendance required at all these events. John and Neville Moray summarized the discussions in their book (Senders & Moray, 1995), but that Norman says does not match his memory of the event. But then again, with all that food and drink, who knows what really happened?

John was interested and expert in almost everything. His home was piled high with a fascinating collection of books on an eclectic array of topics. His professional life was equally eclectic. He was kicked out of Antioch College for his refusal to take the required first-year math course because he said, “I’ve known this stuff since I was 7 and I’ll be damned if I’ll do it again” (Senders & Sellen, 2019). Eventually, he did get an undergraduate degree from Harvard and immediately thereafter started his illustrious career. After jobs in industry and other academic institutions, he became a professor at the University of Toronto, eventually becoming a tenured full professor (remember, he only had an undergraduate degree). After he retired, his Dutch friends helped him assemble four of his publications into a PhD dissertation on “Visual Scanning Processes,” and so, in 1983, he received his PhD from Tilburg University in the Netherlands. This was typical John. Flunk out of freshman year in college for sheer stubbornness. Get an undergraduate degree from a different university, that tiny school on the river in Cambridge, just upstream from MIT. Become a world-renowned expert in Human Factors, a tenured professor at a major research university and then, on approaching retirement, get a PhD.

Retirement for John was of course not really retirement. Well into his 80s he became an expert on healthcare, building on his expertise and foundational work in human error, and showing how lessons learned from fields such as aviation and the nuclear power plant industry could also benefit from the application of principles and methods from Human Factors Engineering. In the course of this, he helped to establish the Institute for Safe Medication Practices in Canada, and later introduced the failure mode and effects analysis (FMEA) technique (Senders & Senders, 2006) into medication safety through the American Institute for Safe Medication Practices. This new endeavor in healthcare also led to a sideline as an expert witness in cases of medical error (as well as trademark infringement, driving error, and numerous other kinds of cases). Later, in his late 80s, he went on to teach law courses at Osgoode Law School in Ontario (at York University), one of the most prestigious law schools in Canada. He even proclaimed, on his 98th birthday, that when he turned 100 and his brain began to slow down, he would become a lawyer.

So how should we write an article in honor of someone like John, “professor of everything”? As authors, “everything” is too large a domain to treat well. Instead, we will focus upon his work on the topics that most concern the three of us: human error, healthcare, and the relationship between these two subjects. As we will describe, though John’s interest in healthcare came rather late in his life, the passion he felt for the topic, and the impact he had on it, was substantial.

## John Senders’ Adventures in Healthcare

John Senders is Professor Emeritus of Engineering at the University of Toronto and Lecturer in Law at York University in Toronto. He has spoken and written on the nature and source of human error since 1976. He is Principal Scientific Consultant to the Institute for Safe Medication Practices (ISMP) and a member of the board of its Canadian offspring (ISMP-Canada). After a routine visit to his physician he unexpectedly found himself on the cardiac treadmill and 10 days later had a quadruple coronary bypass at a major Toronto hospital with an excellent reputation in coronary surgery. He recovered rapidly and is *back at work*. (Senders, 2002): Italics in the original)

In 2002, and at the age of 82, as the quotation above describes, John needed major cardiac surgery. When he entered the hospital, he could not turn off his ability to observe, analyze, and critique his own experience. For most people with a life-threatening ailment and a risky procedure, this would be a time to focus on the personal and to make an attempt to distance the mind from professional distractions. Not John: he turned it into a publication (Senders, 2002).

His first impression following surgery was not technical or medically related, but was the dismay at the mental anguish one experiences, “the dark patches” as he expressed, and how it was not acknowledged. His bunkmate seemed to be experiencing the worst of it, even contemplating suicide. John’s family said that the first night following surgery was “appalling,” improving for the second night to “abominable.” The family only found out later that this was a typical reaction to this kind of surgery. If they had known, it would have been less alarming for them, and for John.

Despite John’s miserable experience, he still applied his expertise in human factors and published his observations in a major medical safety journal (*Quality and Safety in Healthcare*), complete with supporting commentary from family members (Senders, 2002).

The journal article started with the paragraph we quoted at the start of this section. The statement claimed that the hospital had “an excellent reputation in coronary surgery.” That was probably true, but that didn’t stop John (and his family) from noting major flaws. For example, the patient identity cards and card holders were identical to those used at another hospital that were implicated in a blood transfusion error that led to a death. John had been part of an investigation of the earlier case and discovered the defective card holder. He reported the problem in his case with dismay, exclaiming that “Apparently the first hospital had not informed the other hospitals of the danger of the defective card holder.”

In John’s paper, the Senders family reported care issues. One (named as “Family member #2 but who was his son, Stefan) organized the issues into a failure taxonomy including *procurement* (access to a working oxygen regulator), *infrastructure* (bed controls out of reach of patient), *social structure* (doctors unwilling to assist with toileting), and simply *errors* (oxygen line connected to vacuum line, insulin delivered to wrong room, among others). “Family member #1” (his wife, Ann Crichton-Harris) reported errors of having food delivered to the room when there were explicit orders for “nothing by mouth” by the bedside, and on another occasion the food tray was removed without checking if the patient had eaten anything. He had not. The hospital might have a fine reputation for operations (although John was doubtful about even that, given his observations during recovery), but they failed in what is today called “patient experience.”

The journal article is followed by a commentary that was almost as long as the paper, but one that wholeheartedly agreed and amplified the complaints. The writer, Claire Rayner in the UK, started by saying “as one who spends much time working with UK hospital managements, I found comfort in hearing that Canadians—superb though we all know their health service to be—have the same problems that exist here in the UK” and then concluded her comment by stating

A highly informed and demanding (not a pejorative word, but strongly approving in this context) patient like professor Senders improves professional and individual practice hugely for the better. People like him and his family are a priceless resource for any system of health delivery. They should be brought on board as much as possible. (Senders, 2002)

## Defending Against Human Error

From his hospital bed in Toronto, Senders believed that his own “aggressive” self-defense was the final defense against error that could harm him (Senders, 2002). He recruited the hospital staff to do the same by attacking the traditional medical caste system where surgeons, other physicians, nurses, technical staff, and patients were ranked in that order of priority and deference. If they insisted on calling him by his given (first) name, that would be acceptable only if everyone called everyone else by their given names. Because they refused, he insisted on being addressed as Professor Senders.

His insistence on knowing what was to happen to him in advance and in informing the staff of his discomfort made the hospital staff notice him more and become more attentive. Even so, he made sure his family was in attendance in order to supervise and check on the medical treatment he was receiving, something that today is so important that there are now active professions of “hospital sitters” and “health advocates.” For John, back in 2002, this was about setting up various lines of defense against what he believed would be inevitable: that errors would occur, and that accidents would happen.

Looking back on John’s foundational work on human error, it is easy to understand his apprehension. It was not just that he had worked on so many cases of medical error where patients were injured or died, and where medics were blamed and even jailed. Rather, this was due to his basic beliefs about the cause and prevention of errors and accidents.

John’s work on error always distinguished between error and accidents.

From an external viewpoint, an error is a failure to perform an intended action that was appropriate given the circumstances. In my view, an error can only occur if there was or should have been an appropriate intention to act on the basis of a perceived or a remembered state of events; and if the action finally taken was not that which was or should have been intended. (Senders, 1993)

Accidents, on the other hand, he defined as events that are unplanned, unexpected, and undesired. John was always keen to make clear that errors don’t always lead to accidents, and accidents are not always caused by error. After all, many, if not most, errors are detected or fail to have a deleterious outcome. And sometimes accidents are the unhappy confluence of multiple events, including what he referred to as “Acts of God.”

At this point, Norman would have interrupted: “No John, you are wrong in your definition of error. You are defining the error category called ‘slip.’ The other category is a ‘mistake,’ where the intention was carried out perfectly, but it is the intention that is wrong.” John would firmly object to the objection, claiming that his words hadn’t been read with care—after all, he had included “*should have been* intended,” and the two would have a lively debate to the dismay of the onlookers, but thoroughly enjoyed by John and Don.

Not only did John see errors as fundamentally psychological phenomena, but he also strongly believed them to be inevitable, taking to heart the old aphorism “to err is human.” In his view, errors are endogenous, caused by psychological, physiological, or neurological processes inside the actor (Senders, 1993). Following in the footsteps of theoretical work by Norman (1981) and Reason (1990), he also wrote about the various psychological mechanisms that underpinned different kinds of errors, for example (Senders, 1994). However, it is fair to say that of far more interest to him was what he called the *expression* of an error, or the particular way in which an error plays out in a particular situation. In other words, errors can and will occur in any environment, their expression being very different depending on the environment where they occur. So, an error at home in the kitchen may well have undesirable consequences, but that same error in an operating theater will potentially be much worse. As he says,

The expression must depend on what is available to be done in the environment. In a medical setting, an error of substitution may result in a nurse picking up a 2 g prefilled Lidocaine syringe (its *expression*) instead of a 100 mg syringe. In a nuclear power plant, the same error might be expressed by turning off the wrong pump. (Senders, 1994)

Following on from this, John’s work made clear that the circumstances under which an error is expressed not only shape its manifestation, but all kinds of factors (which he called generally “exogenous”) can raise or lower the frequency of errors. Those same factors can raise or lower the likelihood that they will be detected. Here is where John’s research, as well as his work in legal cases of medical error, made the most impact because here is where he showed we can have some degree of control.

## The Need for “Defensive Design”

One such area was errors in medication, working with his friend and colleague Mike Cohen, with whom he would later set up the Institute for Safe Medication Practices (ISMP). Building on work by Davis and Cohen (1981), they analyzed many different factors which increased the frequency of medication errors, ranging from poor handwriting of prescriptions, to ambiguous abbreviations, to poorly designed packaging and nonstandard labeling, to procedural problems. This analysis led to a comprehensive set of recommendations on how to prevent or lower the frequency of medication errors (Cohen et al., 1994). Likewise, he also helped conduct a detailed analysis of anesthetic incidents using one of the first medical incident monitoring systems to be set up (the AIMS or Australian Incident Monitoring System), in this case by proactive group of Australian anesthetists. This research found that almost half of the reports pointed to design deficiencies in anesthetic equipment or the work environment (Sellen et al., 1998). This included problems with the functional design of alarms, tracheal tube design, absorbers, IV drips, and valves, not to mention poor workplace design and set-up of equipment. This work, and much else besides, reflects the approach that John advocated: that of what he liked to call “defensive design” in order to prevent errors in the first place, to aid their detection, or if they occurred, to ameliorate their consequences.

Another recommendation John made was to apply standard methods of risk assessment used in the aerospace, nuclear and defense industries: FMEA. FMEA had long been used to identify all the ways in which a product or system might fail, and then to analyze the possible consequences of those failures. John’s proposal was instead to apply a human error and effects analysis (HEMEA) to healthcare: in short, to assume that whatever can go wrong, will go wrong in relation to the use of any product, piece of equipment, system, or process (Senders & Senders, 2006). This systematic method of examining all possible errors and all possible consequences could be used not only to guide us toward better, defensive design, but would help in post hoc analyses of incidents and accidents. It again reflected his view of the inevitability of human error and human failure and looked toward better design of the whole system as the way forward.

## Defending Against the Healthcare System

John’s insights show the importance of understanding the mechanisms of human error for developing safer medical practice. But coming back to his experience in the hospital, his observations also point out that a lack of systems thinking will inevitably produce an environment where error and accidents flourish. Healthcare is changing, but all too often it is optimizing locally without thinking of the entire picture, the interconnections and large-scale systems that comprise healthcare and hospitals. Instead, the focus is upon individual roles (Swensen et al., 2010). And even when consideration is given to larger units, such as the operation room, emergency department, or intensive care unit, there is little consideration to the nurses, the technical, or the administrative staff, and little understanding that what goes on outside the boundaries of these specialized medical wards are essential to the performance within the wards (Rouse, 2008).

As another example, in 2015, the University of California, San Diego’s hospital system got so many complaints about poor patient service that they reassigned an existing physician to be the “Chief Experience Officer,” who focused entirely upon the patients. One of our authors (Norman) tried—unsuccessfully—to convince him that a focus only upon the patients was wrong. Why? Because it is an interconnected system, and if the physicians, nurses, and staff all had miserable experiences, so too would the patients. It is essential to realize that the hospital is a system with multiple people, multiple stakeholders: all must be addressed. Norman’s effort failed. The Experience Officer said that he was only asked to address the needs of patients. One result was big signs, warnings, and new training (for nurses, not anyone else) on the need for better attention to patients. “Stop talking so much, nurses were told, it annoys the patients.” Maybe so, but perhaps talking is essential to communication as well as for the mental well-being of nurses. The result of the intense focus upon patients was to increase the workload for everyone, and in some cases, make everyone’s jobs more difficult, further diminishing the experience of patients. Meanwhile, the burnout rate among the staff, including the physicians, reached such a high rate that expert consultants were called in to give advice (McKee et al., 2020).

The hospital system—and the newly appointed experience officer—did their best. They required every hospital member to attend a series of presentations about the poor care of patients. Everyone had to attend, from the highest-level administrator to the lowest level staff person (including Norman, although his opinions were never solicited). One of the complaints that everyone had to listen to was given by a patient who was also a professional comedian. He described how he had suddenly encountered severe symptoms that almost completely disabled him. Because he thought he was dying but was living alone and didn’t know whom to call, he painfully climbed into his car and carefully drove to the hospital’s emergency department where he staggered out of the car and stumbled toward the entrance, only to be accosted and told that he couldn’t leave his car there. He had to take it to the parking lot and walk back. “While dying?” he asked himself (and his audience).

Note that the lack of systems thinking even invades the diagnostic and prescription process. All physicians are trained to think of the human body as a complex system. More advanced thinkers also consider the environment, the patient’s work, family activities and arrangements, and eating, sleeping, and activity behaviors. But all this training is lost because of the rise of extreme specialists, where each specialist basically sees the patient through their own particular lens. Few view the patient as a person. As a result, specialists see the ailments they are trained to look for and treat, and then prescribe treatments and drugs that often conflict with the prescriptions and advice of other specialists that are also treating the patient. This sort of separated, disconnected attention to the patients gives rise to a large number of errors. One is over-prescription (McCartney, 2016). Second, is poor communication during shift changes, where special needs, special pre-orders, or even the need for language translators, are often not passed along (Epstein, 2014). The staff is not to blame: they are tired, exhausted, and given little time to do the complex sharing of patient information between shifts. This is one reason that patient advocates are essential. Sometimes patients get forgotten, a situation that Eliah Aronoff-Spencer (an infectious disease specialist) and Norman have called “dropping the patient” (Norman et al., 2018, December 3).

Remember the patient staggering into the emergency ward but being forced to return to his car and park it properly? That’s an example of dropping the patient. How did that happen? Because the emergency department probably thought its concerns were restricted to handling patients admitted into their area. The guards/attendants outside thought that their job was to direct people to the proper location. Parking police were responsible for keeping the area clear. Nobody was taking a view of the whole system: the patient was dropped. This happens at boundaries: when shifts change, or the patient is transferred from one ward to another, or different specialists come in to give advice. Transitions leave gaps, which if not addressed by a systematic analysis of the total system lead to errors, to dropping the patient.

If you want better patient experience, remember, examine the entire system!

John advocated for this level of examination his entire career. Today, we call this way of thinking human-centered design (HCD), which has four fundamental components:

1. Focus upon the people (all the people involved in the system).
2. Solve the fundamental, underlying causes, not the symptoms.
3. Treat the entire system.
4. Every device and procedure need to be prototyped, tested, and refined continually before being put into service.

In the opinion of the authors though, healthcare often fails at all four.

## The Human Factors Profession and The Healthcare “System”

Whereas other industries have a long history of tapping the “scientific discipline concerned with the understanding of interactions among humans and other elements of a system, and the profession that applies theory, principles, data, and other methods to design in order to optimize human well-being and overall system performance” (International Ergonomics Association, 2020), healthcare has been slow to benefit. It has been less than a decade since the U.S. Food and Drug Administration has mandated the use of its methods to determine use error safety of medical devices, let alone addressing system-side issues (Center for Devices & Radiological Health, 2019).

The difficulty here is that many Human Factors professionals are wonderfully prepared to develop procedures and guidelines that are indeed appropriate as well as to analyze existing systems and point out the flaws, but they are seldom able to design or to build new systems. The problem is similar to that of specialists in the healthcare system. Each specialist sees their specialty but leaves to others the piecing together of the recommendations.

We strongly believe that Human Factors cannot be separated from practice. Human Factors professionals must deal with the entire system, which means being a part of the development, of the implementation, and the day-to-day running. No single person can do all of this, but the profession, as a whole, should be involved in all the stages. This is not true today.

Human Factors today is a service organization. It waits to be called upon. This is what has to change.

Human Factors, along with other service organizations such as design, human–computer interaction, and human–systems integration, needs to train its practitioners to rise through the hierarchy of organizations until they reach a position of authority, where they can be involved in all aspects of the system, where they can observe its entire functioning, with an emphasis upon the people, the fundamental issues, and the need to always be assessing performance and making changes where needed. In other words, they need to be working to implement and assess all four stages of the HCD model.

Human Factors and design are two related but somewhat separate disciplines: they shouldn’t be. Both employ similar skills. Both are today relegated to service positions instead of being makers and doers. Both need to change.

Which brings us to John’s fundamental observation of the healthcare “system.” He assumed that safety recommendations, such as those he made with respect to patient identity cards, would be appropriately shared and implemented across the “system.” The use of quotations here is deliberate, because it is difficult to call healthcare a system when it has been described by leading scholars as “a cottage industry of non-integrated, dedicated artisans who eschew standardization” (Swensen et al., 2010).

Unlike other safety-critical industries, healthcare has few formal mechanisms to disseminate safety learnings (Mathews & Pronovost, 2008). In addition, any learnings are largely unenforceable because the system allows a wide range of professional autonomy, where the “professional culture of medicine has deep roots in the mediaeval craft guilds” (Evans et al., 2006).

## Complexity, Unpredictability, and Harm

These issues could be easily attributed to the *complexity* of healthcare, or more specifically healthcare as a *complex adaptive system*. A characteristic of such systems is that it is “composed of *independent agents* whose behavior is based on physical, psychological, or social rules rather than the demands of system dynamics” (Rouse, 2008). All of which would explain why a physician was unwilling to help with John’s toileting when asked, and the seeming indifference of the food service staff. Still another characteristic of such systems is that there is often no one in-charge. No one has the authority or resources to design the system, resulting in *self-organization*, whereby a “system” emerges without any guidance or agreed upon structure or cohesion. Although this can occasionally elicit valuable innovations, more often it leads to a lack of cohesion and appropriate communication channels. Serendipitous self-organization might be fine for creativity and dealing with novel situations quickly, but it is not an appropriate way to develop organizational structures for long-term treatment on matters of life, death, or permanent injuries (Toomari & Cafazzo, 2019).

In his writings, John argues that the design of hospitals is often detrimental to their own goals. The American Institute of Medicine’s (IoM) report stated that the “simple rules and minimum specifications” of most hospital procedures’ deviant behaviors arise out of what appears to be a necessity to get the work done, with the “writing (of) operating rules that are never followed precisely” (Institute of Medicine, 2001). In his paper “On the Complexity of Medical Devices and Systems,” Senders (2006) suggested that in many hospitals, rule creation is done under the “unimaginative” assumption that the created rules will be followed, rather than to consider people’s propensity to deviate and ultimately err.

He argued that the unpredictability and harm that occurs far too often in healthcare can be mitigated, as errors are often predictable using a Human Factors engineering lens. It requires an effort to create internal *engineering* complexity in order to reduce the external *surface* (user) complexity. “As less and less skill, judgment, and intelligence are required (or needed) at the user-system interface of a device or system, more and more complexity is required behind the interface to provide the operational intelligence required by the goals’’ (Senders, 2006). This wholly counters the advice of the seminal IoM guidance provided nearly two decades ago, and it is not surprising that harm due to medical error continues to haunt the industry (Pellegrini, 2020; Sutcliffe, 2019).

John, of course, did not believe in following rules that he considered misguided or just plain stupid. He praised those who deliberately “misused” medical devices (Senders, 2001), but he carefully distinguished between misuse due to ignorance and that which was the result of expert knowledge by those who knew the device well and were “skilled in systematically imaginative behavior.” He called these people “creative mischief makers” suggesting that this practice of experts should be studied and used to change the regulations (obviously considering himself to be in that category). We add that this is the method of “lead user innovation,” strongly advocated by Eric Von Hippel (1988).

## Final Words

This tribute to John was written during the first few months of the global pandemic of 2020. At this point, we don’t know how it ends, but we do know the panic that ensued at the start. Healthcare “systems” were attempting to increase capacity in anticipation of a surge of COVID-19 patients that could overwhelm hospitals and force them to make awful moral decisions about those they would treat and those they would not.

In Toronto, Human Factors professionals were asked to quickly evaluate the safety of ventilators from little known manufacturers in a matter of hours, as procurement decisions needed to be made immediately. They were asked to comment on the use of ventilator splitter schemes (Sommer et al., 1994) and improvised open-source ventilator designs (Armani et al., 2020). Decision makers didn’t wait for the usually impeccably detailed Human Factors reports to be written. Conclusions were relayed by text message. Then came the challenges of their personal protective equipment (PPE), and the inevitability of thousands of healthcare workers who would err in the doffing and donning of their masks, gloves, and gowns, potentially contaminating themselves (Herlihey et al., 2016). Outbreaks in hospitals ensued (CBC News, 2020), and Human Factors staff was called in to find out why. Training materials, workflows, and mitigations needed to be prepared in a fraction of the time that would be expected under normal circumstances.

The hospital leadership needed solutions, not reports with recommendations. Had the Toronto Human Factors professionals stopped at analysis, they would have failed their colleagues and the patients. Increasingly, Human Factors professionals are turned to as solution providers, not just problem identifiers. Design must become synonymous with Human Factors, just as design must embrace the science and rigor of Human Factors (Cafazzo & St-Cyr, 2012). Hence, the continued importance of the profession advancing beyond the wisdom that John and others have provided us over the last 50 years.

Those of us who knew John miss his wisdom, wit, and most of all, his opinions and insights, delivered always in his deep, sonorous voice. For our part, and for the sake of healthcare, we are grateful that his voice still resonates today.

## Key Points

- John Senders excelled in the study of human error and its implications for healthcare systems.
- He taught that “… as less and less skill, judgment, and intelligence are required (or needed) at the user-system interface of a device or system, more and more complexity is required behind the interface to provide the operational intelligence required by the goals.’’
- He suggested that in many hospitals, rule creation is done under the “unimaginative” assumption that the created rules will be followed, rather than to consider people’s propensity to deviate and ultimately err.
- Well into his 80s, he became an expert on healthcare, building on his expertise and foundational work in human error, and showing how lessons learned from fields such as aviation and the nuclear power plant industry could also benefit from the application of principles and methods from Human Factors Engineering.
